# Association between Pneumonia, Fracture, Stroke, Heart Attack and Other Hospitalizations with Changes in Mobility Disability and Gait Speed in Older Adults

**DOI:** 10.3390/jcm10173802

**Published:** 2021-08-25

**Authors:** Joshua D. Brown, Reiko Sato, John E. Morley

**Affiliations:** 1 Rx-Epitome, LLC, Gainesville, FL 32606, USA; 2 Patient and Health Impact, Pfizer Inc., Collegeville, PA 19426, USA; reiko.sato@pfizer.com; 3 Division of Geriatric Medicine, Saint Louis University School of Medicine, St. Louis, MO 63104, USA; john.morley@health.slu.edu

**Keywords:** pneumonia, heart attack, stroke, fracture, hospitalization, physical activity, physical functioning, older adults, gait speed, mobility

## Abstract

Pathophysiological changes after acute hospitalizations may influence physical functioning in older adults, which can lead to disability and loss of independence. This study evaluated the association between pneumonia, fracture, heart attack, stroke, and other hospitalizations with major mobility disability (MMD) and gait speed. This was a secondary analysis of the Lifestyle Interventions and Independence for Elders (LIFE) Study, which was conducted across eight sites during 2010–2013 with longitudinal follow-up for 1635 individuals over an average of 2.6 years. Participants included adults ≥70 years old with pre-existing mobility limitations randomized to a physical activity intervention or a health education control arm. Hospitalizations were recorded via self-report and adjudicated by medical reviewers. MMD was measured by the inability to complete a 400 m walk test, or other proxies, as a binary outcome. Gait speed was recorded during the walk test in meters per second (m/s) and measured on a linear scale. Mixed-effects repeated measures regression adjusted for baseline demographics, comorbid conditions, and frailty. Among the 1635 participants, there were 1458 hospitalizations, which included 80 (5.5% of all hospitalizations) cases of pneumonia, 92 (6.3%) hospitalized fractures, 87 (6.0%) heart attacks, and 61 (4.2%) strokes. In the short-term measurement period immediately following hospitalization (1 day to 6 months), stroke (OR = 3.98 (3.41–4.54)) had the strongest association with MMD followed by fracture (OR = 3.03 (2.54–3.52)), pneumonia (OR = 2.76 (2.23–3.30)), and heart attack (OR = 2.03 (1.52–2.53)). Associations with long-term (6–12 months after) MMD were decreased or not significant for all causes. Pneumonia, fracture, stroke, and other hospitalizations were associated with short-term relative gait speed changes between −4.8% up to −19.5%, and only fracture was associated with long-term changes. Hospitalizations for pneumonia, heart attack, stroke, and fractures were associated with short-term decreases in mobility in older adults. Older adults may be at risk for decreased mobility and disability following acute hospitalizations, with the magnitude determined by the cause of the precipitating event.

## 1. Introduction

The onset of physical functioning disability can be described as a progressive process or be characterized by an abrupt, catastrophic onset [1,2]. Catastrophic onsets are often precipitated by intervening illnesses or injuries, with the strongest associations involving hospitalization events [2]. There is a significant risk of functional decline after precipitating health events, which can ultimately impact quality of life and independence for older adults [2,3]. Decreased physical functioning in older adults is a strong predictor of future mortality [3,4]. From a patient and societal burden perspective, physical functioning decline also increases healthcare utilization and expenditures [5]. Preventing or slowing this decline can have a significant societal impact and improve the lives of older adults, their families, and caregivers [6].

A key to understanding the link between intervening health events and the onset of disability is the timing of assessments after the intervening event and the physiological changes that may lead to the disability. Existing studies evaluating precipitating health events have measured changes over large intervals (e.g., 12 or 18 months), which may not capture acute changes in physical functioning [2,7]. The physiological changes leading to disability are dynamic and can be transient. Thus, assessing physical functioning changes more distal from an intervening health event (e.g., 6–18 months after an event) may not fully capture the acute changes in physical functioning following an event [2,7,8,9]. Further, study outcomes often include terminal measures of disability (e.g., needing assistance in activities of daily living) rather than the physical functioning changes that may lead to this disability.

This study evaluated the associations between hospitalizations and major mobility disability (MMD) and gait speed as measures of physical functioning. Mobility, defined as the ability to walk without assistance, is critical to an older adult’s ability to remain in a community setting and function independently [10,11]. MMD has been linked to increased morbidity, institutionalization, and mortality in older adults [12,13,14,15]. Using robust and objectively measured outcomes from the Lifestyle Interventions and Independence for Elders (LIFE) Study [16,17], the immediate and long-term associations of pneumonia, fracture, heart attack, stroke, and other hospitalizations on MMD and gait speed were assessed. These hospitalization subtypes (pneumonia, fracture, heart attack, and stroke) were specifically analyzed as they are acute onset events (compared to pre-existing conditions) that often lead to pathophysiological changes that may impact physical functioning [18,19,20,21,22]. These conditions are associated with high readmission rates and healthcare costs with significant morbidity and mortality risk in older adults [23]. The study hypothesis was that these hospitalization subtypes would be associated with significant decrements in physical functioning in both the short-term and long-term measurement periods, compared to other hospitalizations.

## 2. Experimental Section

The LIFE Study was a multi-center, single-blind, parallel randomized trial conducted across eight centers in the United States between February 2010 and December 2013 [17]. The study protocol was approved by the institutional review boards of each institution. Written informed consent was obtained from all study participants. The trial was monitored by a data and safety monitoring board appointed by the National Institute on Aging. The LIFE Study was registered with www.clinicaltrials.gov (accessed on 12 July 2021) before participant enrollment in the trial (NCT01072500). LIFE Study data for this study were provided by the National Institute on Aging’s Aging Research Biobank after ethical approval by an independent review board.

Details of the study design, rationale, and characteristics of the full study population are described elsewhere [17,24]. Participants were eligible for the trial who were 70–89 years of age, scored <10 on the Short Physical Performance Battery (SPPB), were sedentary with ≤125 min of activity per week, and were able to complete the 400 m walk test within 15 min without sitting, leaning or without assistance.

### 2.1. Intervention

Details of the study interventions were previously published [17,25]. The physical activity (PA) intervention involved walking, with a goal of 150 min per week, strength, flexibility, and balance training. The intervention included attendance at two center-based visits per week and home-based activity three to four times per week for the study duration. The PA sessions were individualized and progressed toward 30 min of walking daily at moderate intensity, 10 min of primarily lower-extremity strength training using ankle weights (2 sets of 10 repetitions), 10 min of balance training, and large muscle group flexibility exercises.

The health education (HE) intervention included weekly educational workshops during the first 26 weeks and then monthly sessions after that. Workshops included topics relevant to older adults, such as negotiating the health care system effectively, how to travel safely, preventive services and screenings recommended at different ages, where to go for reliable health information, nutrition, etc. The workshops did not include any PA topics. The program also included a 5 to 10 min instructor-led program of gentle upper-extremity stretching or flexibility exercises.

### 2.2. Follow-Up Visits and Outcome Assessment

Details of MMD ascertainment were reported previously [16]. Participants were asked to walk 400 m at their usual pace, and MMD was defined as the inability to complete the walk within 15 min without sitting and without the help of another person or walker. When MMD could not be objectively measured, an alternative adjudication of the outcome was based on objective inability to walk 4 m in less than 10 s, or self-, proxy-, or medical record-reported inability to walk across a room. Gait speed was measured based on the completion time of the 400 m walk test or from the SPPB. Participants were assessed every 6 months at clinic visits. Home, telephone, and proxy assessments were attempted if participants could not return to the clinic.

### 2.3. Hospitalization Events

Participants or their proxies self-reported all healthcare utilization and reasons for that utilization since the last clinical visit. That is, the events would have occurred between 1 day and 6 months before the current outcome assessment visit. For hospitalizations, medical records were extracted for adjudication of medical diagnoses and procedures performed. Medical record assessors were blinded to the intervention assignment, and Medical Officers at each clinical site used standardized criteria (Medical Dictionary for Regulatory Activities, MedDRA) to categorize reasons for hospitalization. Hospitalizations were categorized as pneumonia, fracture (any location), heart attack, stroke (ischemic or hemorrhagic), and other (all other causes, e.g., cancer, pulmonary disorders, surgeries/procedures). Hospitalization for pneumonia, fracture, heart attack, and stroke were selected as acute conditions potentially representing new-onset events, distinguished from other hospitalizations involving planned (e.g., surgery) or chronic conditions (e.g., chronic pulmonary disease exacerbation, cancer).

### 2.4. Statistical Analyses

Cohort demographics and baseline characteristics were described for the overall LIFE Study cohort by hospitalization category. A repeated measures framework for each LIFE Study participant was used and included all follow-up assessments recorded. Hospitalization exposures were recorded with dummy variables. MMD was recorded as a binary outcome and analyzed via repeated measures logistic regression with fixed effects for baseline variables and random effects within individuals. Gait speed was analyzed as a continuous linear outcome with similar mixed effects models. The analyses separately focused on two exposure–outcome relationships: (1) the “short-term” effects represented by the assessment period immediately following the hospitalization (between 1 day and 6 months); and (2) the “long-term” effects represented by the subsequent assessment period (between 6 and 12 months) after hospitalization. These models were adjusted for baseline demographics, and functional assessments. Baseline covariates modeled as fixed effects included intervention assignment, age (per year), sex, SPPB ≤ 7 or >7, race (White, Black, Other), education (≥high school), smoking status (current, former, never), and medical history (cardiovascular disease, diabetes, stroke, cancer, broken bone, pneumonia, chronic lung disease), prior hospitalization, baseline gait speed, and quintiles of a deficit accumulation frailty index [26]. Odds ratios and 95% confidence intervals were reported from the MMD analysis and the adjusted, relative percent change reported from the gait speed analysis. The reference group for all relative measures was those LIFE Study participants with no hospitalization events. All analyses were conducted in SAS Enterprise Guide v8.1 with an alpha = 0.05 for all analyses.

## 3. Results

Among the 1635 participants randomized during the study period, there was an average of 2.6 years of follow-up time with a loss to follow-up of 4% annually. There was a total of 1458 hospitalizations, which included 80 (5.5% of all hospitalizations) cases of pneumonia, 92 (6.3%) hospitalized fractures, 87 (6.0%) heart attacks, and 61 (4.2%) strokes. Baseline characteristics of the LIFE Study cohort are shown in Table 1.

**Figure 1:**
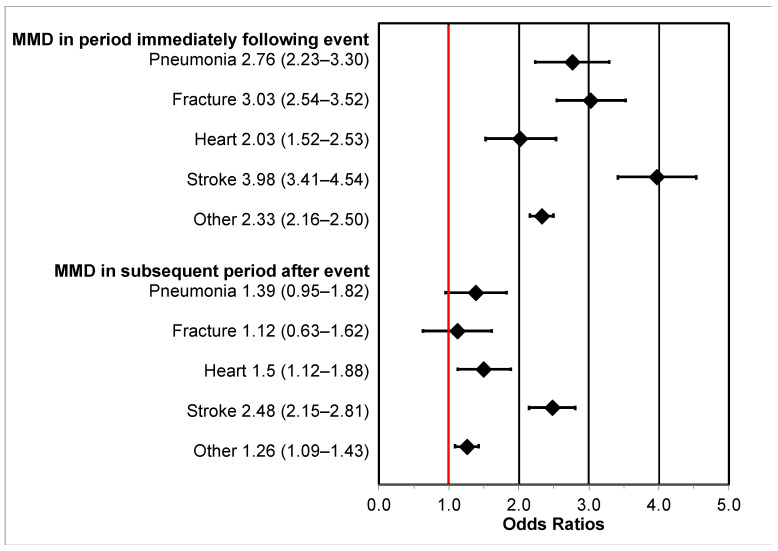
Adjusted odds ratios (OR) and 95% confidence intervals (95% CI) for the association between intervening hospitalization events and major mobility disability (MMD) in the assessment period immediately following hospitalization (“short-term” MMD; 1 day to 6 months after hospitalization) and the subsequent assessment period (“long-term” MMD; 6–12 months after hospitalization). The reference group is those without hospitalization and the “Other” group consists of hospitalizations of any cause. The red vertical line represents the null value.

Associations between hospitalizations and MMD are shown in Figure 1. The association between other causes of hospitalizations and MMD immediately following the event was 2.3 times higher compared to those without hospitalization (OR = 2.33 (2.16–2.50)). Specific hospitalization types were also associated with immediate MMD including pneumonia (OR = 2.76 (2.23–3.30)), fracture (OR = 3.03 (2.54–3.52)), heart attack (OR = 2.03 (1.52–2.53)), and stroke (OR = 3.98 (2.41–4.54)).

Long-term MMD, i.e., lasting from 6 to 12 months after the event, was associated with heart attacks (OR = 1.5 (1.12–1.88)), stroke (OR = 2.45 (2.15–2.81)), and other hospitalizations (OR = 1.26 (1.09–1.43)). Associations of long-term MMD with pneumonia (OR = 1.39 (0.95–1.82)) and fracture (OR = 1.12 (0.63–1.62)) hospitalizations were not statistically significant.

**Figure 2:**
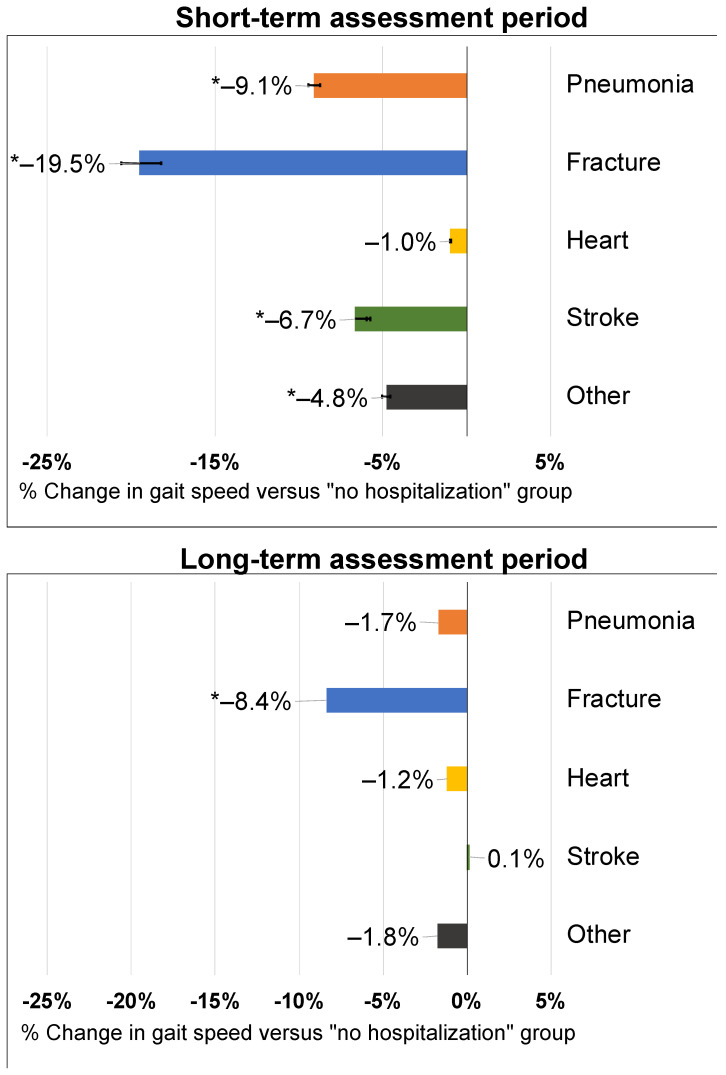
Adjusted percent change in short-term and long-term gait speed after an intervening hospitalization event. Labels indicate the point estimate and the *p*-value for the comparison to the “No event” group. Data labels represent point estimates. Asterisk (*) denotes statistical significance versus those without a hospitalization.

Gait speed reductions were observed for all hospitalization types (Figure 2). Fracture hospitalizations had the largest, regression-adjusted percent change in gait speed of −19.5% (−21.9 to −17.5%; *p* < 0.001) compared to those without hospitalization. Pneumonia hospitalizations were associated with a significant decrease in gait speed of −9.1% (−10.2% to −8.3%; *p* < 0.001) as were stroke hospitalizations (−6.7% (−6.5% to −6.8%); *p* < 0.001) and all other hospitalizations (−4.8% (−5.4% to −4.3%); *p* < 0.001). Heart attacks were not associated with a significant change in gait speed.

Long-term changes in gait speed were only significantly associated with prior fracture hospitalizations (−8.4% (−9.7% to −7.3%)). All other hospitalization types were not associated with long-term gait speed changes (Figure 2).

## 4. Discussion

These findings show the associations between hospitalization events with changes to physical functioning using robust, objectively measured outcomes from the LIFE Study. Results show significant associations between intervening hospitalizations and MMD for all hospitalizations and stronger associations with pneumonia, fracture, and stroke-related hospitalizations in the short term. Over the long term, these associations were attenuated overall but showed a remaining strong association between long-term MMD and stroke.

Our findings correspond to prior analyses of the LIFE Study, which evaluated the impact of intervening hospitalization events on the stability of the physical activity intervention and the association of these hospitalizations with future MMD [27,28]. The prior findings suggested a stronger association (HR~3.3) between hospitalizations and MMD and no influence by or on the PA intervention [28]. The difference in association shown by our study here is due to the prior study evaluating the exposure–outcome relationship over the entire follow-up period in a survival analytic framework versus the current study, which focused on the immediate and subsequent assessment periods following hospitalization using repeated measures. Thus, these different results provide varying insights into the short- and longer-term impacts of intervening hospitalization events.

Prior research has compared pneumonia to other acute, hospitalized conditions such as heart attack, stroke, and serious fractures [23,29], which are all leading causes of morbidity and mortality in older adults [30]. In fact, these were the most frequent adverse events and reasons for hospitalization among the LIFE Study cohort [16,27]. However, pneumonia is unique amongst this group of conditions, especially heart attack and stroke, as it can also be managed on an outpatient basis. Approximately 60% of the 1.3 million community-acquired pneumonia episodes that occur annually in the United States in older adults will be outpatient [31]. Recent work has shown the impact of inpatient versus outpatient pneumonia events on similar outcomes of MMD and gait speed [32]. While inpatient pneumonia events have a more substantial effect on MMD and gait speed than outpatient events, the overall burden of pneumonia should be considered in context for both outpatient and inpatient events.

Our hypothesis that intervening hospitalizations would be associated with functional decrements was confirmed but lessened by weaker or no associations with long-term changes for MMD and gait speed. These findings suggest two possible scenarios. For one, decrements in physical functioning due to intervening health events may be dependent on the specific cause of the event (e.g., pneumonia, stroke, heart attack, or fracture), and even catastrophic disability may not be permanent, with physical functioning measures being recoverable within 6–12 months post-event. A second scenario is that MMD and gait speed are not sufficient measures of physical functioning to capture the effects of intervening events such as hospitalizations. An additional implication of these findings is that if functional status is measured 6–12 (or longer) months after an event, the associations observed may be reduced. Thus, for both clinical and research purposes, functional assessments more proximal to hospitalization may better capture the impact of these intervening health events.

### Limitations

This study is strengthened by a clinically relevant cohort of older adults and frequent objective measures of mobility outcomes. However, the cohort was explicitly selected based on the LIFE Study inclusion and exclusion criteria; thus, the results may not be generalizable to the overall older adult population. There was potential for recall bias for hospitalization events as these relied first on self-report. This would bias the observed results towards the null effect if hospitalizations were underreported, meaning the results of this study were conservative. The cohort was selected based on the LIFE Study criteria and was undergoing a physical activity intervention or participating in a control arm, which may limit the generalizability of these results to the overall older adult population and calls for additional research in more generalizable cohorts. The timing of each hospitalization was not recorded in the publicly available LIFE Study data; thus, immediate assessment periods were analyzed with hospitalizations occurring 1 day to 6 months prior and long-term assessment periods analyzed with hospitalizations occurring 6–12 months prior. Given the evidence that the impact of intervening hospitalizations wanes over time, this approach lost information on more acute effects of intervening events (e.g., within the first 30 days), which deserves additional research. While MMD and gait speed were most commonly assessed via the 400 m walk test, proxies including a shorter walk test or by reported inability to walk independently were also used. In these scenarios, it may be possible that individuals passing a shorter walk test are misclassified if they would have failed the longer walk. This limitation most likely led to an underestimation of the hospitalization effect, given more individuals may be classified as not having MMD and having improved gait speeds when, in fact, these assessments may have been failed or lower. Lastly, while all exposures were hospitalizations, the severity of each condition can vary significantly but could not be assessed in this study, given the nature of the public LIFE Study data.

## 5. Conclusions

Intervening hospitalization events were associated with immediate decrements in physical functioning represented by MMD (inability to complete a 400 m walk) and gait speed. These effects decreased over time but persisted for select conditions up to 12 months after the event. These results suggest that acute physical disability occurs with intervening events but may be recoverable.

## Figures and Tables

**Table 1:** Baseline demographics, medical history, and functional status of the LIFE Study cohort stratified by overall and by those with select acute hospitalization subtypes. All variables are reported as N and % unless otherwise (*) specified.

	Precipitating Hospitalization Subtype									
	Pneumonia N = 80		Heart Attack N = 87		Stroke N = 61		Fracture N = 92		Other N = 1147	
Demographics										
Age, years *	80	5	80	4	80	5	82	5	79	5
Female	55	68.4%	51	59.0%	37	60.7%	69	75.4%	772	67.3%
Race										
Black	11	13.2%	11	12.1%	13	21.3%	1	1.5%	184	16.0%
Other	6	7.9%	4	4.8%	1	1.6%	3	2.9%	71	6.2%
White	63	79.0%	72	83.1%	47	77.1%	88	95.7%	892	77.8%
Education ≥ high school	46	57.9%	59	67.5%	38	62.3%	67	72.5%	770	67.1%
Smoking										
Former	40	50.0%	41	47.0%	21	34.4%	36	39.1%	551	48.0%
Current	2	2.6%	2	2.4%	3	4.9%	3	2.9%	25	2.2%
Prior hospitalization ≤6 months prior to intervention	13	15.8%	11	13.1%	7	11.7%	8	8.8%	133	11.6%
Intervention assignment	40	50.0%	43	49.4%	34	55.7%	40	43.5%	592	51.6%
Comorbidities/Medical History										
Cardiovascular Disease	34	42.1%	37	42.2%	27	44.3%	25	27.5%	427	37.2%
Stroke	8	10.5%	9	10.7%	10	16.4%	3	2.9%	100	8.7%
Cancer	27	34.2%	27	31.0%	19	31.2%	19	20.3%	303	26.4%
Diabetes	13	15.8%	27	31.3%	21	34.4%	21	23.2%	331	28.9%
Broke bone	29	36.8%	33	38.0%	26	42.6%	41	44.9%	448	39.1%
Pneumonia	36	45.0%	33	38.0%	27	44.3%	37	40.2%	437	38.1%
Chronic Lung Disease	29	36.8%	14	15.7%	11	18.0%	13	14.5%	218	19.0%
Physical Functioning										
SPPB ≤ 7	47	65.8%	41	47.0%	25	41.0%	57	62.3%	530	46.2%
SPPB *	6.9	1.6	7.2	1.8	7.5	1.4	6.8	1.7	7.3	1.7
400 m Walk gait speed (m/s) *	0.7	0.18	0.71	0.23	0.64	0.20	0.56	0.23	0.71	0.22
Deficit Accumulation Frailty Index *	32	7	33	7	31	7	30	6	32	7

Abbreviations: SPPB = Short Physical Performance Battery; LIFE Study = Lifestyle Interventions and Independence for Elders Study. * Continuous variable; mean and standard deviation reported.

## Data Availability

Data are publicly available through the AgingResearchBiobank.
